# Age affects antibody levels and anthelmintic treatment efficacy in a wild rodent

**DOI:** 10.1016/j.ijppaw.2019.03.004

**Published:** 2019-03-14

**Authors:** Melanie Clerc, Simon A. Babayan, Andy Fenton, Amy B. Pedersen

**Affiliations:** a Institute of Evolutionary Biology and Centre for Immunity, Infection and Evolution, School of Biological Sciences, University of Edinburgh, Edinburgh, EH9 3FL, UK; b MRC Centre for Inflammation Research, Queen´s Medical Research Institute, University of Edinburgh UK, EH16 4TJ, UK; c Institute of Biodiversity, Animal Health & Comparative Medicine, University of Glasgow, Glasgow, G12 8QQ, UK; d Institute of Integrative Biology, University of Liverpool, Liverpool, L69 7ZB, UK

**Keywords:** Anthelmintic treatment, Age-dependent immunity, *Eimeria hungaryensis*, *Heligmosomoides polygyrus*, Within-host interaction, Wild immunology

## Abstract

The role of the host immune system in determining parasite burdens and mediating within-host parasite interactions has traditionally been studied in highly controlled laboratory conditions. This does, however, not reflect the diversity of individuals living in nature, which is often characterised by significant variation in host demography, such as host age, sex, and infection history. Whilst studies using wild hosts and parasites are beginning to give insights into the complex relationships between immunity, parasites and host demography, the cause-and-effect relationships often remain unknown due to a lack of high resolution, longitudinal data. We investigated the infection dynamics of two interacting gastrointestinal parasites of wild wood mice (*Apodemus sylvaticus*), the nematode *Heligmosomoides polygyrus* and the coccidian *Eimeria hungaryensis,* in order to assess the links between infection, coinfection, and the immunological dynamics of two antibodies (IgG1 and IgA). In an anthelmintic treatment experiment, mice were given a single oral dose of an anthelmintic treatment, or control dose, and then subsequently followed longitudinally over a period of 7–15 days to measure parasite burdens and antibody levels. Anthelmintic treatment successfully reduced burdens of *H. polygyrus*, but had no significant impact on *E. hungaryensis*. Treatment efficacy was driven by host age, with adult mice showing stronger reductions in burdens compared to younger mice. We also found that the relationship between *H. polygyrus*-specific IgG1 and nematode burden changed from positive in young mice to negative in adult mice. Our results highlight that a key host demographic factor like age could account for large parts of the variation in nematode burden and nematode-specific antibody levels observed in a naturally infected host population, possibly due to different immune responses in young vs. old animals. Given the variable success in community-wide de-worming programmes in animals and humans, accounting for the age-structure of a population could increase overall efficacy.

## Highlights

• Anthelmintic treatment reveals strong force of infection for *H. polygyrus* in wild wood mice.
• Anthelmintic treatment is more successful in younger compared to older mice.
• Relationship between IgG1 and *H. polygyrus* burden reverts with host age.

## Graphical abstract

**Figure:**
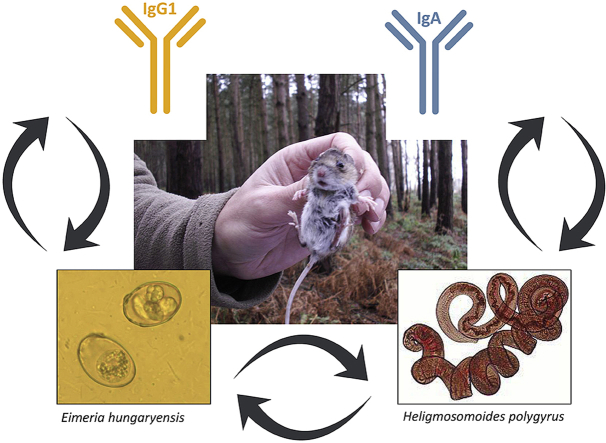

## 1 Introduction

In the wild, animals and humans vary greatly in key demographic characteristics such as sex, age, reproductive status and infection history (Babayan et al., 2011). Variation in these factors can potentially influence the strength and polarisation of a host's immune response (Nussey et al., 2012), leading to differences in disease susceptibility, severity, morbidity, and mortality between individuals (Hayward et al., 2014; Budischak et al., 2015). However, our understanding of the importance of various demographic factors in shaping disease burden and susceptibility to parasitic infections by creating variation in the host's immune response is limited due to the narrow demographic range and genetic diversity of studies carried out in the lab (e.g. 6-week old inbred mice, Bordon, 2016), and the limited resemblance lab mice share with humans and animals in the wild (Mestas and Hughes, 2004; Beura et al., 2016). Hence, understanding the interplay between the immune response and host demography in shaping parasite infections (prevalence and burden) requires the integration of both immunological and ecological data in an easily accessible natural system.

Even though still rare, studies measuring immune parameters in the wild allow unique insight into the dynamic relationships between immunity, parasite infection and coinfection, and host health (Pedersen and Babayan, 2011). Studies of humans in endemic disease areas have indicated important associations between immunity and infection burdens, for example (Hegewald et al., 2015) found that children infected with different nematode and protozoan parasites show a mixed pro-inflammatory and regulatory immune cell profile, and that while IL-27 and IL-33 levels increased with the number of coinfecting parasite species, IL-10 levels remained constant (Hegewald et al., 2015). Further, children infected with *Schistosoma spp.*, *Entamoeba*, and *Necator americanus* exhibited reduced inflammatory responses but increased type 2 responsiveness to antigen stimulation after anthelmintic treatment (Hamm et al., 2009). In wild Soay sheep, variation in antibody levels was found to correlate with over-winter survival, such that high levels of IgG1 produced against nematodes were positively associated with survival, whereas high levels of IgM produced against generic non-self antigens such as keyhole limpet haemocyanin (KLH) were negatively associated with survival (Nussey et al., 2014). On the other hand, a study linking nematode coinfection with host demography, haematological parameters and investment in lymphocyte defence in wild African buffalo only found very few weak associations in male buffalos, and no significant associations in female buffaloes (Budischak et al., 2012). These and other studies highlight that there is a great deal of variation in immune parameters amongst individuals in the wild, and that these immune parameters might be key predictors of host health and parasite infection dynamics (Nussey et al., 2014). However, more data on fine-scale patterns of both parasite and immune dynamics are needed to draw reliable conclusions about the interplay between immune parameters, host demography and variation in parasite infections.

Crucially though, studies in the wild are often unable to discriminate between predictors of protective immunity and immune markers of infection. Due to time lags between parasite exposure, immune activation and build up, and parasite clearance, it can be hard to disentangle cause from effect based on snapshot measures of immune and parasite status (i.e., whether measured immune parameters are indicative of protection against current or future infections, generating a negative relationship between immune and parasite measures, or whether immune parameters are simply triggered by parasitic infections, generating a positive relationship between them (Babayan et al., 2011; Pedersen and Babayan, 2011)). A key factor influencing this relationship between observed levels of host immune parameters and parasite burden is host age, due to intrinsic changes in how the immune system responds to parasite infection over time (Humphreys and Grencis, 2002), the accumulation of previous parasite exposure (DeVeale et al., 2004), and how chronic parasitic infections affect the ageing immune system (Babayan et al., 2018). Individuals are likely to accumulate both parasite exposure and immune memory as they age. Young individuals can have both immature immune systems and infrequent parasitic exposure, whereas older individuals may have limited novel parasite exposure but fully developed innate and adaptive immune responses, and could have potentially started to experience immunosenescence (Ginaldi et al., 2001). This means that a single immune parameter can potentially change from an immune marker in young individuals, to a predictor of immunity in older individuals, making it challenging to infer the cause-and-effect relationships between age, immune function, and infection history in natural systems. To overcome this, we require studies that measure all of these aspects and repeatedly sample wild animals at a high temporal resolution across a range of host ages, ideally coupled with experimental perturbations to disrupt the interconnectedness of host age, parasite burden and immune parameters.

Here, we present the results of an experimental study using wild wood mice (*Apodemus sylvaticus*) to elucidate the relationships between host age, natural levels of general and parasite-specific antibodies and naturally-occurring infections with two interacting gastrointestinal parasites.

The wood mice in our system are commonly infected by the coccidian *Eimeria hungaryensis* and the nematode *Heligmosomoides polygyrus*. *H. polygyrus* is closely related to the lab-adapted species *H. bakeri* (Cable et al., 2006; Behnke and Harris, 2010), which has been used extensively in laboratory studies as a model of gastrointestinal human helminth infections (Maizels et al., 2012; Reynolds et al., 2012). This has revealed that parasite-specific antibodies play a key role in immunity to *H. polygyrus* in mice. Specifically, *H. polygyrus*-specific IgG1 can trap and immobilize tissue-dwelling larvae, promote phagocytosis, and wound-healing via alternatively activating macrophages (Esser-von Bieren et al., 2013; Hewitson et al., 2015). Furthermore, we previously found that treating wild mice with an anthelmintic drug (Ivermectin) not only reduced the prevalence and intensity of *H. polygyrus* infections, but also significantly increased the intensity of *E. hungaryensis* in coinfected hosts, demonstrating a negative antagonistic within-host interaction between the two parasites (Knowles et al., 2013). Interestingly, we also showed in a cross-sectional experiment that mice infected with *Eimeria* spp. had both lower levels of *H. polygyrus*-specific IgG1 and total faecal IgA antibodies (Clerc et al., 2018). *Eimeria hungaryensis* infects cells of the host's duodenum, in which it undergoes several rounds of asexual replication before entering a sexual cycle, leaving the infected cell to burst with newly formed oocysts. IgA is the most prevalent antibody at mucosal surfaces, where high-affinity IgA antibodies protect from microparasite infections such as *Eimeria* spp., while low-affinity IgA antibodies regulate commensal bacterial densities via immune exclusion (Cerutti and Rescigno, 2008; Macpherson et al., 2012). The precise role of IgA in anti-*Eimeria* immunity has not yet been elucidated but anti-schizont and anti-sporozoite activities of IgA have been shown during *Eimeria* infections in chickens (Davis et al., 1978; Trees et al., 1989). Overall, this accessible wild system is ideally suited to study the interplay between parasitic infections (both single and coinfection), variation in host demography and variation in key antibody levels as a readout for the host immune response.

We performed a longitudinal anthelmintic treatment experiment in a wild population of wood mice to investigate i) the short-term effect of anthelmintic treatment on *H. polygyrus* infection probability and burden, as well as the associated response against the non-drug target parasite *E. hungaryensis*, ii) the effect of anthelmintic treatment on specific and general antibody dynamics, iii) the cause-and-effect relationship between antibody levels and parasite infection, and iv) the effect of host age on the relationship between antibody levels and parasite infection. Using a high trapping frequency, we were able to closely follow immune and infection dynamics upon disturbance of a natural coinfection/interaction system, giving unique insights into the relationship between immune function, host demographic variation and parasitic infection.

## 2 Methods

All animal work was done under Home Office Project Licence 70/8543.

### 2.1 Field work

Our experiments took place in Callendar Woods, Falkirk, Scotland (55.99° N, 3.77° W). We performed the experiment over three distinct sessions, each session lasting 8 weeks: Session 1: July to August 2014, Session 2: October to November 2014 and Session 3: June to July 2015. In 2014, the experiment consisted of one large grid in which 2 Sherman live traps (H. B. Sherman 2 × 2.5 × 6.5 inch folding trap, Tallahassee, FL, USA) were placed every 10 m in a 130 × 80 m rectangle (total 256 traps). In 2015, the large grid was split into two smaller grids that were separated by ∼10 m, and an additional third grid was added ∼50 m north of the other two grids. All three grids were 70 × 70 m in size and consisted of a total of 294 traps. Traps were baited with bedding, mixed seeds, mealworms and a piece of carrot the day before a trapping night. The following morning, traps were checked for the presence of animals. At first capture, each wood mouse received a unique passive induced transponder tag (PIT) injected subcutaneously in the scruff (AVID FriendChip), which allowed individual identification of mice at subsequent captures. Further, all captured mice were randomly selected to receive either a single oral dose of a combination of anthelmintic drugs: Ivermectin at 100 mg/kg (Wahid and Behnke, 1993) and Pyrantel at 9.4 mg/kg (Wahid et al., 1989), or water as a control. Combining these two drugs enabled us to simultaneously target both adult (Pyrantel) and the larval stages (Ivermectin) of *H. polygyrus* (Wahid et al., 1989). For each mouse at each capture, we recorded spatial location of capture, sex, reproductive status (females: perforated vagina, pregnant or lactating, males: non-reproductive, testes descended or scrotal), body weight, body length, and body condition (dorsal and pelvic fat reserves, each scored from 1 to 5). We also checked each mouse for the presence of ectoparasites such as mites, fleas or ticks. At one capture each week (including first capture), we took a blood sample via puncture of the facial vein. The blood was spun down in the lab for 10 min at 12000 rpm and sera and blood pellet were stored individually at −80 °C. Faecal samples were collected from the pre-sterilized traps that each animal was captured in: 2–3 pellets were dry-frozen at −80 °C for total faecal IgA ELISAs, whereas the rest was stored in 10% Formalin at 4 °C for faecal egg counts (Dryden et al., 2005). We determined parasite infection (infected/uninfected) and burdens (number of eggs/oocysts per 1 g of faeces) by counting parasite transmission stages after salt floatation using a microscope at either 10× or 40× magnification (see Knowles et al., 2012).

From previous studies in this system, we know that anthelmintic drugs only reduce *H. polygyrus* burden for about 10–16 days (see Knowles et al., 2012). Thus, we aimed to monitor each mouse over a time-span of 15 days. At the beginning of each experiment session (first two weeks), trapping took place daily in order to catch, tag and treat as many mice in the population as possible. After this period, trapping frequency changed to three times per week until the end of the session (8 weeks). Thus, mice could potentially be trapped up to 10 times within the 15 days window if they first appeared at the beginning of a session and had a high trapping fidelity. However, since trapping fidelity can be low for some animals, we started culling mice from 7 days post-treatment onwards in order to maximise sample size and toe ensure that we capture the mice when drug treatment was still effective (up to 14 days). If an animal was caught after the 15 days period, it was still culled and the data collected up to 15 days post-treatment was used in the analysis, but the parasitological/immunological data collected after 15 days post-treatment (faecal egg/oocyst counts, antibody levels) were excluded from the analysis, as anthelmintic treatment is no longer effective (Knowles et al., 2013). Mice were culled via cervical dislocation and a terminal blood sample was obtained via cardiac puncture. For each animal, we visually screened the gastrointestinal tract (small intestine, caecum and colon) under a dissecting microscope to detect the presence and quantity of intestinal nematodes. We used dried eye lens weight (ELW; grams) as a quantitative measurement of host age. This metric has been well established as a reliable proxy for host age due to the consistently strong curvilinear relationship between ELW and age in various species of small mammals of known age (Morris, 1972; Thomas and Bellis, 1980; Hardy et al., 1983; Rowe et al., 1985; Tanikawa, 1993; Burlet et al., 2010). Furthermore, the relationship is also stable to variation in other body characteristics such as body mass and length (Thomas and Bellis, 1980). Both eyes were collected and stored in 10% Formalin at 4 °C for at least 4 weeks. After separating the eye lenses from the surrounding tissue, they were dried at 56 °C overnight and weighted in pairs to the nearest mg.

### 2.2 Immunological methods

We focussed on two antibodies in this study: Total faecal IgA and *H. polygyrus*-specific IgG1. Importantly, these can be measured in wood mice using laboratory mouse reagents (whereas reagents for other immune markers, such as key cytokines, have yet to be developed in this non-model organism), making IgA and IgG1 the most appropriate immune markers to study in these animals. To measure *H. polygyrus*-specific IgG1, we coated plates (Nunc™ MicroWell™ 96-Well Microplates) with 0.05 μg *H. polygyrus* excretory-secretory antigen obtained from cultured adult worms (HES, supplied by R. M. Maizels) in 50 μl carbonate buffer overnight at 4 °C. Non-specific binding sites were blocked with Tris-buffered Saline (TBS) containing 4% Bovine Serum Albumin (BSA) at 37 °C for 2 h. Two-fold serial dilution of blood serum were prepared in cluster tubes containing TBS-1% BSA, starting at 1:100. A serum-sample of artificially *H. polygyrus*-infected lab *M. musculus* was added to each plate as a positive control (supplied by R. M. Maizels). After plates were washed with TBS-0.1% Tween 20, sample dilutions were added to the plates (50 μl per well) and incubated overnight at 4 °C. After washing, 50 μl goat anti-mouse IgG1-HRP (Southern Biotech, Lot J6908-MC69), diluted 1:2000 in TBS-1%BSA was added to each well and incubated at 37 °C for 1 h in the dark. Plates were washed 4 times with TBS-Tween 20 and 2 times with _d_H2O, before 50 μl Tetramethylbenzidine (TMB) solution was added to each well. Plates were immediately covered with tinfoil and the enzymatic reaction was let to develop for 7 min. The reaction was stopped with 50 μl 0.18 M Sulphuric acid and absorbance at 450 nm was measured. Cut-off values were calculated per plate as mean absorbance of blank wells plus 3 times the standard deviation. The sample titre was determined as the denominator of the lowest sample dilution step that showed absorbance greater than the cut-off value.

For the faecal IgA ELISA, faecal extracts were prepared by adding 3 times the sample volume of protease inhibitor solution to the sample (Complete Mini Protease Inhibitor Tablets, Roche, Cat No.:11836153001). Samples were let to incubate for 1 h at room temperature, after which samples were centrifuged at 12′000 rpm for 5min and supernatant containing IgA removed. ELISA plates were then coated with 0.1 μg of unlabelled goat anti-mouse IgA (Southern Biotech, Lot H7912-S233) in 50 μl carbonate buffer. Non-specific binding sites were blocked with TBS containing 4% BSA at 37 °C for 2 h. Faecal extracts were diluted 1:100 in cluster tubes containing TBS-1% BSA and added to the plates as triplicates at 50 μl per well. Two twofold serial dilutions of standard antibody (Purified mouse IgA, κ isotype control, BD Pharmingen, Lot 3039828) at 50 μl per well, were added to each plate. Plates were incubated overnight at 4 °C. After washing, 50 μl goat anti-mouse IgA-HRP (Southern Biotech, Lot G4512-V522D), diluted 1:4′000 in TBS-1% BSA was added to each well and incubated at 37 °C for 1 h in the dark. Plates were washed 4 times with TBS-Tween and 2 times with _d_H2O, before 50 μl TMB solution was added to each well. Plates were immediately covered with tinfoil and enzymatic reaction was let to develop for 7 min. The reaction was stopped with 50 μl 0.18 M Sulphuric acid and absorbance at 450 nm was measured. Sample concentrations of total faecal IgA was determined using the software provided by elisaanalysis.com, by fitting 4-parameter logistic regression to standard curves.

### 2.3 Statistical analysis

**Fig. S1:**
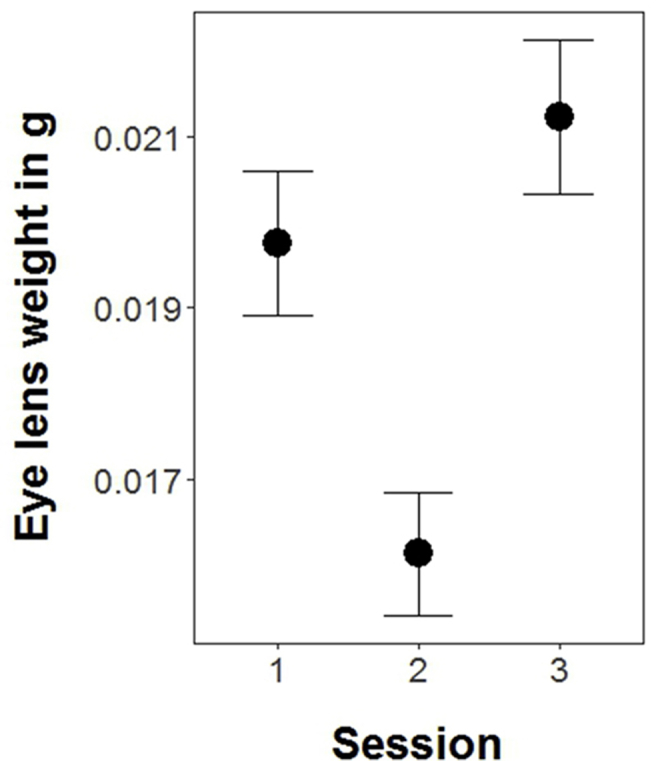
Age differences between experimental sessions. ELW was used as a proxy for host age.

All statistical analyses were performed using R version 3.2.2 (R Development Core Team (2013), www.r-project.org). Mice showed marked age-differences between sessions (Supplementary figure S1), with animals captured in session 2 (autumn) having significantly lighter eye lenses than animals in sessions 1 and 3 (ANOVA, session F_2,86_ = 10.51, p < 0.0001). This suggested that animals captured in the autumn were on average younger than those captured in late spring/early summer. This corresponds with the breeding season of wild wood mice, i.e. the spring mice are likely to be the ones that survived the winter, whereas the autumn mice are new young-of-the-year animals (Montgomery, 1989). Hence, the variables “age” and “session” were highly correlated, and preliminary analysis showed that this caused issues when both were simultaneously used in a model. Because “session” could not be fitted as a random effect (only three levels), we only used “age” in our subsequent models as it gave us a better resolution of the population variation in age due to the continuous nature of this variable.

### 2.4 Temporal dynamics of treatment effects

We fit four individual models to assess the effect of time after anthelmintic treatment on either (i) *H. polygyrus* burden (HP model), (ii) *E. hungaryensis* burden (EH model), (iii) *H. polygyrus*-specific IgG1 titre (IgG1 model) and (iv) total faecal IgA concentration (IgA model). All response variables were continuous, but because *H. polygyrus* and *E. hungaryensis* burdens showed a high frequency of zeros (uninfected animals), we used a Tweedie distribution with a point-mass at zero (Dunn and Smyth, 2008), whereas we used a Gaussian distributions for the IgG1 and IgA models. Each of the four models tested the effects of the following factors: host sex (male or female), age (continuous; scaled eye lens weight (ELW)) and year (2014 or 2015), with animal ID fit as a random effect to account for multiple measurements per individual mouse. Further, we included *E. hungaryensis* infection at first capture (factor; infected or uninfected) in the HP model, and *H. polygyrus* infection at first capture (factor; infected or uninfected) in the EH model. In the IgG1 and the IgA models, we included both *E. hungaryensis* and *H. polygyrus* infection at first capture. We used generalized additive mixed effect models (GAMMs) using the *gamm*() function in the “mgcv” R package (Wood, 2011), which enabled us to include a smooth-term for treatment group (treated or untreated) by time post-treatment in the models. This allowed for non-linear relationships between treatment and the response variables to be fitted over time, without prior assumption of the shape of this relationship. Best-fitting models were estimated by stepwise backward exclusion of non-significant terms (cut-off p-value was 0.05), beginning with interaction terms, and compared using Akaike's information criterion (AIC).

### 2.5 Directionality between antibody levels and parasite burden

To assess the causal relationship between antibody levels and parasite burdens, we tested these two hypotheses: 1) antibody levels at first capture affect post-treatment parasite burdens, or 2) parasite burdens at first capture affect post-treatment antibody levels. To test these hypotheses, we constructed four GLM models with either post-treatment parasite burdens (two models) or post-treatment antibody levels (two models) as the response variable. We defined post-treatment as the values of the response variable between 7 and 15 days post-treatment (at day of cull for most animals). We then tested the effect of antibody levels at first capture on post-treatment parasite burdens, and vice versa. For example, the model explaining variation in post-treatment *H. polygyrus* burden included IgG1 and IgA levels at first capture as predictors, whereas the model explaining variation in post-treatment IgA levels included *H. polygyrus* and *E. hungaryensis* burdens at first capture as predictor variables. In all models, we included the following predictors: host sex, age, anthelmintic treatment (factor; treated or control) and year. Further, we fit interaction terms between age (continuous; ELW) and either parasite burden or antibody levels at first capture in each model to account for the possibility of host age driving the variation in either parasite burdens or antibody levels at first capture. We also included interaction terms between age and treatment in each model to account for the possibility of age-specific effects of anthelmintic treatment on the response variable. *H. polygyrus* and *E. hungaryensis* burden were again best described by a Tweedie distribution with a point-mass at zero, and antibody levels by a Gaussian distribution. Best fitting models were estimated by stepwise backward exclusion of non-significant terms (cut-off p-value was 0.05), beginning with interaction terms, and compared using AIC.

## 3 Results

In total, we captured 89 mice (33 females, 56 males): 25 in session 1, 30 in session 2 and 34 in session 3. Mice were captured on average 4 times over the 15 days experimental period (total n captures = 350, range between 1 and 10 captures per mouse). We did not find a significant difference in recapture rates between control and anthelmintic-treated mice (control = 5.1 ± 0.17 SE, treatment = 5.4 ± 0.18 SE captures; two-sample *t*-test p = 0.206).

We gave anthelmintic treatment to 39 animals while 50 animals received control treatment (water). Initial prevalence for *H. polygyrus* was 58%, and 36% for *E. hungaryensis*. However, after treatment, *H. polygyrus* prevalence was reduced to 28% in treated mice, while it increased to 83% in control animals. *E. hungaryensis* prevalence decreased to 26% in treated mice and 29% in control mice.

### 3.1 Short-term effects of treatment

**Fig. 1:**
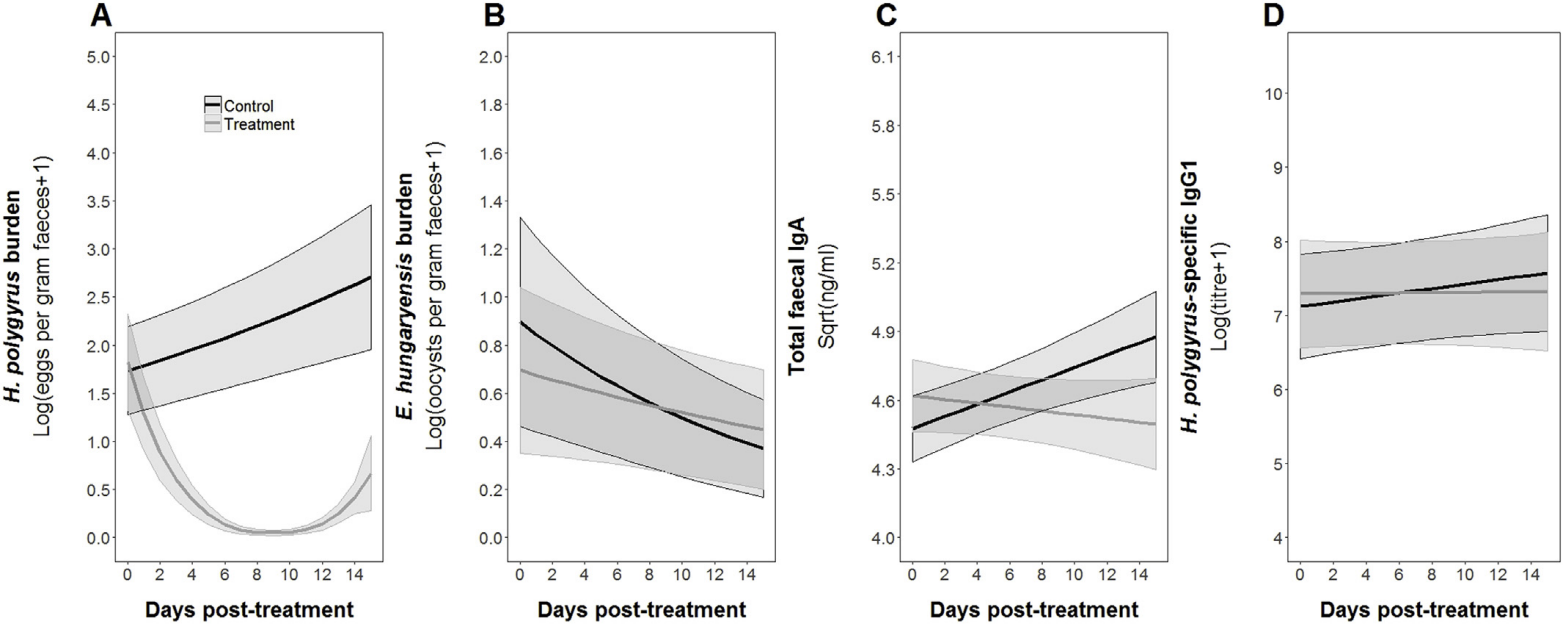
Dynamics of A) *H. polygyrus* burden, B) *E. hungaryensis* burden, C) total faecal IgA and D) *H. polygyrus*-specific IgG1 over the experimental period as predicted by minimal GAMM models. Red lines represent model estimates for animals treated with anthelmintic, black lines represent model estimates for control animals. Shaded areas represent estimated standard errors.

Using a dynamic GAMM modelling approach, we tested the short-term effects of anthelmintic treatment on parasite burdens and antibody levels. We found that anthelmintic treatment had an immediate and strong effect on the burden of *H. polygyrus*, which decreased following treatment until ∼10 days, after which it started to increase again (Table 1, Fig. 1A). Counter to our previous observations (Knowles et al. 2013), anthelmintic treatment did not result in a subsequent increase in *E. hungaryensis* burden (Table 1, Fig. 1B). Parasite burdens were also significantly different between the two years, with lower overall burdens in 2015 compared to 2014 (Table 1). For the two antibodies, total faecal IgA significantly increased over time in control animals (Table 1, Fig. 1C), whereas IgA levels in treated mice stayed constant over the course of the experiment. Further, faecal IgA was positively associated with *H. polygyrus* at first capture infection (Table 1). Lastly, while *H. polygyrus*-specific IgG1 titre did not change over time of the experiment, it did increase with host age (Table 1, Fig. 1D).

**Table 1:** Results for minimal GAMM models on *H. polygyrus* burden, *E. hungaryensis* burden, total faecal IgA and *H. polygyrus*-specific IgG1.

Model covariates	*H. polygyrus* burden	*E. hungaryensis* burden	Total faecal IgA	*H. polygyrus*-specific IgG1
Sex (male)				
Age				1.56, p < 0.0001 ***, df = 83
Initial *Eh* infection (infected)		NA		
Initial *Hp* infection (infected)	NA		0.44, **p** = 0.022 *, df = 84	
*Hp*-specific IgG1			NA	NA
Total faecal IgA		−0.04, **p** = 0.020 *, df = 101	NA	NA
Year (2015)	−0.51, **p** = 0.012 *, df = 84	−0.63, **p** = 0.035 *, df = 84		1.11, p = 0.051, df = 83
Treatment (Ivermectin)	−1.41, p < 0.0001 ***, df = 84	−0.24, p = 0.399, df = 84	−0.39, **p** = 0.042 *, df = 84	−0.48, p = 0.359, df = 83
Ivermectin x days post treatment	NA, p < 0.0001 ***, df = 2.3	NA, p = 0.623, df = 1	NA, p = 0.694, df = 1	NA, p = 0.436, df = 1
Control x days post treatment	NA, p = 0.056, df = 1	NA, p = 0.122, df = 1	NA, **p** = 0.022 *, df = 1	NA, p = 0.404, df = 1

Each column represents a single model, each row represents a model covariate. Each cell contains the covariate estimate and p value. Comparison levels for factors are given in brackets. Cells containing NA represent covariates that were not included in the starting model, while empty cells represent covariates that were not retained in the final model after model reduction. *Hp* stands for *H. polygyrus*, *Eh* stands for *E. hungaryensis*, and x denotes an interaction. ****p < 0.001, **p < 0.01, *p < 0.05, ˙ p < 0.1.

### 3.2 Directionality of interaction between antibody levels and parasite burden

**Fig. 2:**
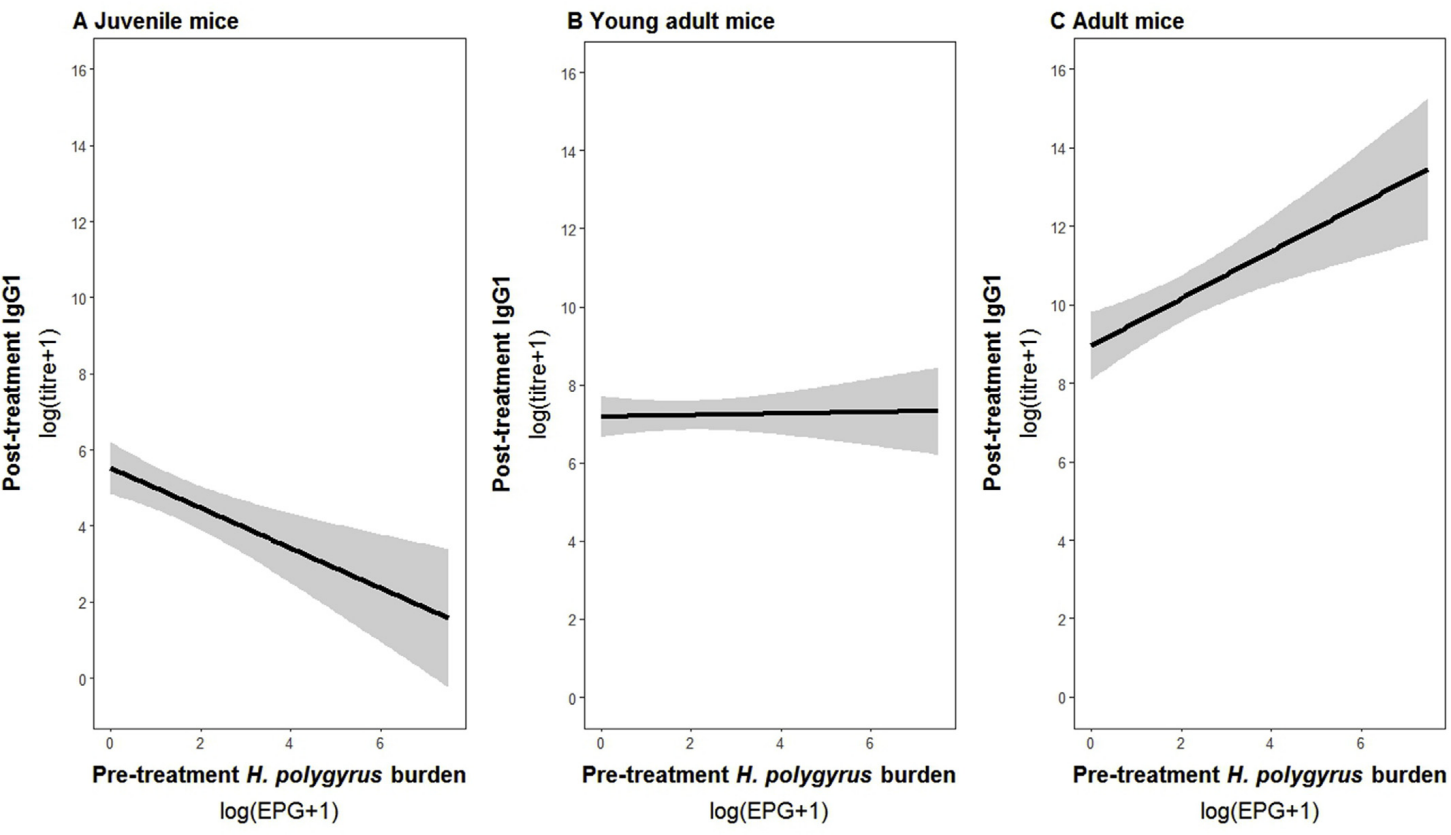
Relationship between mouse age (scaled ELW), treatment and post-treatment *H. polygyrus* burden as predicted by GLM model. The red line represents model estimates for treated animals, black lines represent model estimates for control animals. Shaded areas represent estimated standard errors. Points represent raw data.

**Fig. 3:**
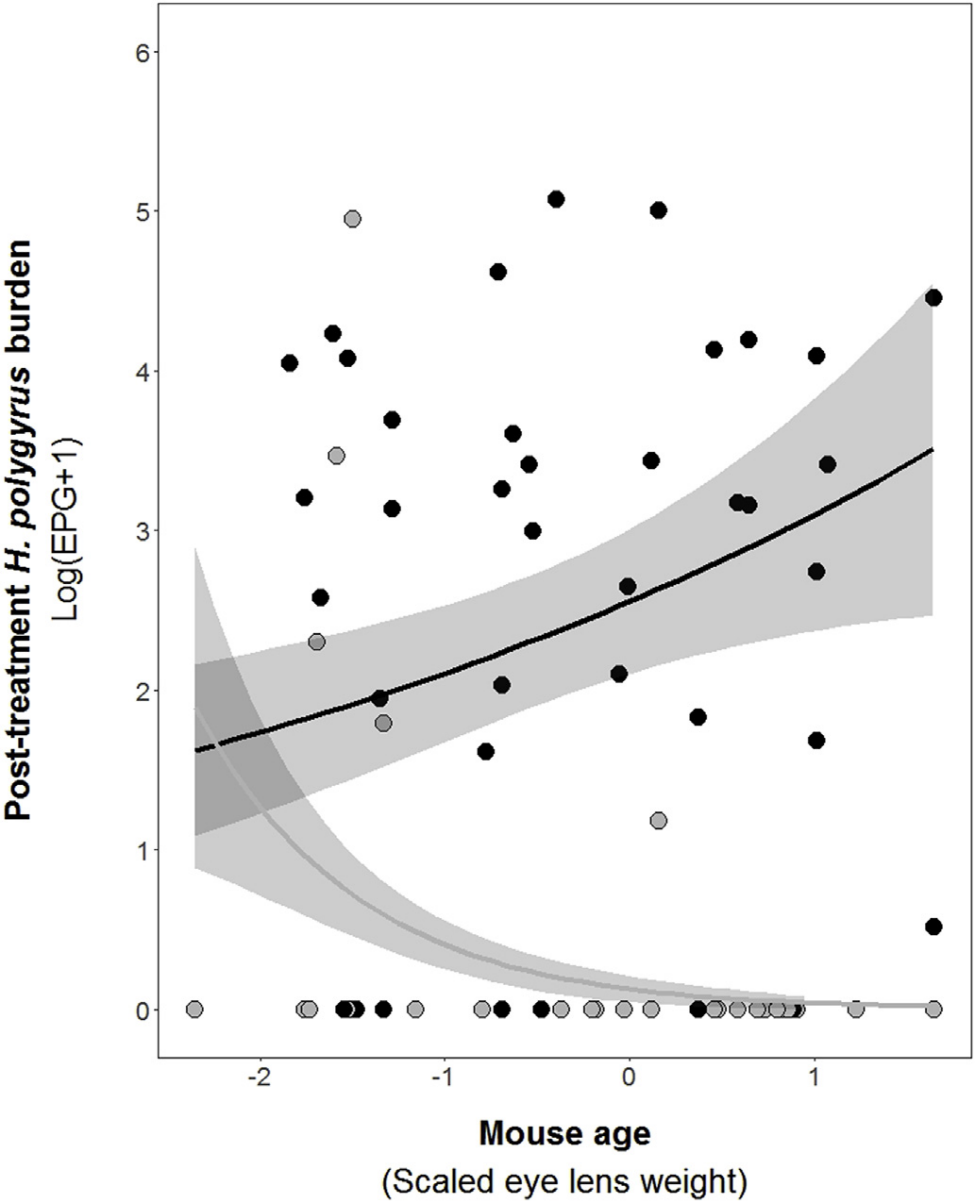
Predicted relationship between pre-treatment *H. polygyrus* burden and post-treatment *H. polygyrus*-specific IgG1 titre for A) juvenile mice, B) young adult mice and C) adult mice. ELW was split into equal thirds to group mice into juvenile (9.2 μg-15.8 μg), young adult (15.8 μg-22.4 μg) or adult mice (22.4 μg-29.0 μg).

**Fig. S2:**
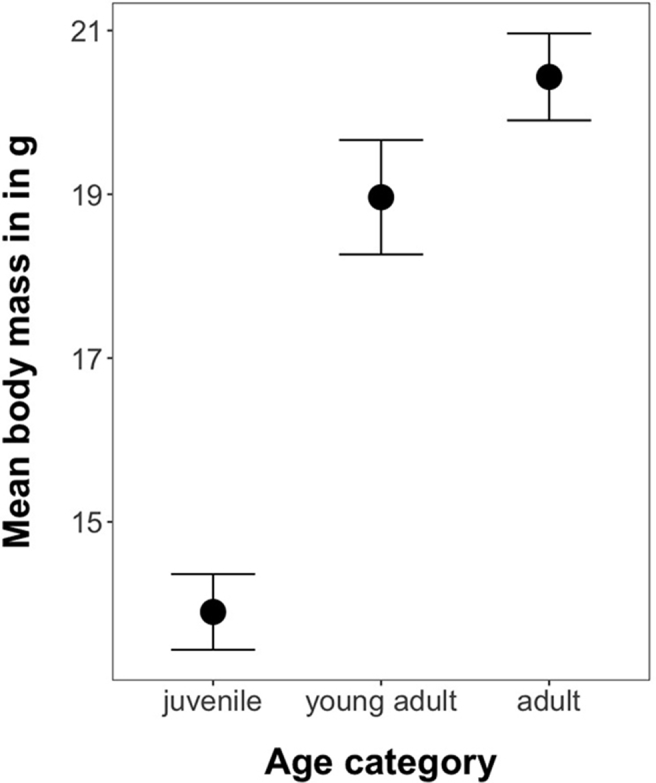
Mean (+/− Standard error) differences in body mass between age categories.

To disentangle the cause and effect relationships between parasite-specific antibody production and parasite burdens, we tested whether antibody levels at first capture could predict subsequent parasite burdens, or vice versa. First, we found a significant relationship between *H. polygyrus* burden at first capture and post-treatment IgG1 titres (day 7–15). Interestingly, this relationship was dependent on host age (ELW). The range in ELW in our populations was 9.2 μg–29.0 μg and thus ELW was a continuous variable in all models. Here, we divided this range in ELW into equal thirds to display the results in age groups: (i) juvenile mice (9.2 μg-15.8 μg), (ii) young adult mice as (15.8 μg-22.4 μg), and (iii) adult mice (22.4 μg-29.0 μg; Fig. 2). These age categories also matched the mean body weights we measured (Fig. S2). In juvenile mice, high *H. polygyrus* burden at first capture was associated with low post-treatment *H. polygyrus*-specific IgG1 titres. This negative relationship flattened out in young adult mice, and actually reversed in adult mice, where high *H. polygyrus* burdens at first capture were associated with high post-treatment IgG1 levels (Table 2, Fig. 2). We also found a significant age-dependent relationship between treatment and post-treatment *H. polygyrus* burden: adult mice treated with the anthelmintic had subsequently lower *H. polygyrus* burdens than adult untreated mice (Fig. 3, Table 2). However, in juvenile mice, treatment had little effect on *H. polygyrus* burdens 15 days post-treatment (Fig. 3, Table 2). Post-treatment *E. hungaryensis* burden was neither explained by the demographic nor immune parameters used in our model. In the case of post-treatment IgA levels, we only found a slight trend towards an increase in IgA levels with age (Table 2).

**Table 2:** Results from minimal GLM models on post-treatment values of *H. polygyrus* burden, *E. hungaryensis* burden, *H. polygyrus*-specific IgG1 and total faecal IgA levels.

Model covariates	*H. polygyrus*-specific IgG1	*H. polygyrus* burden	*E. hungaryensis* burden	Total faecal IgA
Sex (male)				
Age	0.74, p = 0.100, df = 75	0.13, p = 0.344, p = 71		0.30, **p** = **0.092 ˙**, p = 78
Year (2015)	1.01, p = 0.152, df = 73	−0.63, **p** = 0.023 *, p = 72		
*Eh* burden T_0_	0.001, p = 0.992, df = 74	NA	NA	
*Hp* burden T_0_	0.12, p = 0.510, df = 76	NA	NA	0.11, p = 0.151, p = 77
IgG1 T_0_	NA			NA
IgA T_0_	NA			NA
Treatment (Ivermectin)		−2.87, p < 0.0001 ***, p = 70		−0.46, p = 0.120, p = 76
Age x Treatment		−1.13, **p** = 0.002 **, p = 69		
Age x *Hp* T_0_	0.32, **p** = **0.051** ˙, p = 72	NA	NA	−0.11, p = 0.138, p = 75
Age x *Eh* T_0_	0.17, p = 0.178, p = 71	NA	NA	
Age x IgG1 T_0_	NA			NA
Age x IgA T_0_	NA			NA

Each column represents a single model, each row represents a model covariate. Each cell contains the covariate estimate and p value. Comparison levels for factors are given in brackets. Cells containing NA represent covariates that were not included in the starting model, while empty cells represent covariates that were not retained in the final model after model reduction. *Hp* stands for *H. polygyrus*, *Eh* stands for *E. hungaryensis,* and x denotes an interaction. ****p < 0.001, **p < 0.01, *p < 0.05, ˙ p < 0.10.*14.*

## 4 Discussion

Using a longitudinal anthelmintic treatment experiment in a wild wood mice population, we sought to understand the relationship between general and parasite-specific antibody levels, the burdens of two interacting gastrointestinal parasites, and variation in host demographic factors. We showed a strong and immediate effect of anthelmintic treatment on *H. polygyrus* burdens. However, treated mice reverted to their pre-treatment egg shedding patterns within 10 days, highlighting the strong force of helminth infection in nature (Speich et al., 2016). Further, we showed that *H. polygyrus* burdens at first capture determined subsequent *H. polygyrus*-specific IgG1 levels in an age-dependent manner, demonstrating a dynamic relationship between parasite infection, immunity and host age. Finally, host age also affected a mouse's susceptibility to helminth infection, as *H. polygyrus* burdens were reduced following treatment in adult mice, but not in juvenile wild mice. Taken together, our results highlight the force of infection experienced by mice in the wild, and we showed how one antibody can change from an immune marker to a predictor of protective immunity depending on mouse age.

The use of anthelmintic treatment, in combination with a high trapping frequency (3–5 nights per week) meant that we could monitor the short-term responses of coinfecting parasites and general and helminth-specific antibodies following anthelmintic treatment on a fine time scale. This revealed that short-term reduction of *H. polygyrus* burden occurred almost immediately after drug administration, and burdens remained low until around 10 days post-treatment. After this period, *H. polygyrus* burdens began to rise again. Since it takes around 8–10 days for *H. polygyrus* L3 larvae (infective stage) to develop into adult worms in the host intestinal tissue (Valanparambil et al., 2014), the eggs observed 10 days after treatment were likely produced either by female worms that survived treatment and/or newly emerged adult worms that were the product of reinfection shortly after treatment. Counter to our expectations based on the results from Knowles et al. (2013), we did not observe an increase in *E. hungaryensis* burden following the removal/reduction of *H. polygyrus* after anthelmintic treatment. However, *E. hungaryensis* burdens in that earlier study were higher (range 0–181 000 oocysts per gram faeces, mean 2876 oocysts per gram faeces) than in the current study (range 0–213 135 oocysts per gram faeces, mean 1714 oocyst per gram faeces). On the contrary, *H. polygyrus* burdens were higher in this study (range 0–1828 eggs per gram faeces, mean 46 eggs per gram faeces) compared to Knowles et al. (2013) (range 0–563 eggs per gram faeces, mean 33 eggs per gram faeces). This could suggest that the interaction between *H. polygyrus* and *E. hungaryensis* could be density-dependent (e.g. Fenton, 2013), where *H. polygyrus* only has a negative effect on *E. hungaryensis* when they are both at a sufficiently high enough density. Such density-dependent effects of parasite competition are consistent with both resource- and immune-mediated interactions (Griffiths et al., 2015), and have been shown for coinfections between a bacterial and a fungal parasite infection in the water flea *Daphnia magna* (Ebert et al., 2000), and between larvae of three different helminth species infecting the crustacean *Paracalliope fluviatilis* (Lagrue and Poulin, 2008). This highlights that not only is it important to study the mechanism(s) underlying parasite interactions, but also the qualitative and quantitative aspects of infections, as this will ultimately have a big impact for optimising treatment strategies.

We further investigated the cause-and-effect relationships between antibody levels and parasite infections. Previously, we found a negative effect of the presence of *Eimeria* spp. on both *H. polygyrus*-specific IgG1 and total IgA, but it was not clear whether *Eimeria* spp. was the cause of those low antibody levels, or whether mice with low antibody levels were more susceptible to *Eimeria* spp. infection (Clerc et al., 2018). Here, we could not find additional evidence that supported either of these two hypotheses, as initial *E. hungaryensis* burdens were not associated with either post-treatment parasite-specific IgG1 or IgA, or vice versa. As described above, it is possible that *E. hungaryensis* needs to be at a high enough density in order to have a measurable effect on antibody levels. On the other hand, we did find that *H. polygyrus* burden at first capture affected post-treatment levels of *H. polygyrus*-specific IgG1, whereas we did not find any effect of antibody levels at first capture on post-treatment *H. polygyrus* burden. Further, the relationship between *H. polygyrus* burden at first capture and post-treatment *H. polygyrus*-specific IgG1 levels was dependent on age: In juvenile mice, post-treatment IgG1 levels were inversely related to pre-treatment *H. polygyrus* burden, irrespective of treatment. This may reflect the variation in *H. polygyrus* exposure amongst juvenile mice, meaning that they differ in the degree to which they already have established protective immunity. Specifically, those juveniles with high post-treatment IgG1 and low pre-treatment *H. polygyrus* burden have likely been exposed more often and hence have already established higher levels of IgG1-mediated protective immunity compared to those juveniles with low post-treatment IgG1 and high *H. polygyrus* burden at first capture. This agrees with *H. polygyrus*-specific IgG1 dynamics found in laboratory mice, where only multiple *H. polygyrus* infections led to the production of affinity-maturated parasite-specific antibodies that had the capacity to protect the host against re-infection and high burdens of adult worms (McCoy et al., 2008; Hewitson et al., 2015). In contrast, we observed that high pre-treatment *H. polygyrus* burdens in adult mice quickly (within 15 days) triggered high post-treatment IgG1 levels, likely due to higher baseline IgG1 levels as a consequence of previous infections, suggesting that with increasing age, the role of IgG1 changes from an immune effector in young mice to an immune marker in adult mice. This is a rare demonstration of how the relationship between antibody and parasite infection changes over the course of a host's life, suggesting that it is crucial to not only consider a wider range of host ages in controlled laboratory experiments, but also to account for population age-structure when assessing the meaning of immune measurements assessed from wild animals.

Importantly, we showed that variation in host age is associated with variation in the strength of the host's immune response, which in turn significantly affects the efficacy of Ivermectin treatment. Age-dependent variation in treatment efficacy has also been found in human de-worming studies that include pre-school aged children, where treatment efficacy varies depending on child age, treatment dose, helminth species, infection intensity and the time point when efficacy was assessed (Albonico et al., 2008). Further, Jackson et al. (2014) showed that wild field vole males transitioned from a resistant to a tolerant phenotype towards macroparasite infection as they age (Jackson et al., 2014). Here, we found that in juvenile mice, post-treatment *H. polygyrus* burdens did not differ between treated and control mice. In contrast, treatment efficacy increased with increasing host age and was very effective in adult mice. Ivermectin acts by binding to glutamate-gated chloride channels, which are not present in vertebrates. In doing so, Ivermectin directly interferes with nematode motility, feeding and reproduction, without affecting the hosts immune response (Laing et al., 2017). Therefore, we suggest that Ivermectin acts upon *H. polygyrus* in a consistent fashion over a mouse's lifetime, and these age-related changes are due differences in the strength of protective immunity. Lab studies have shown that at least one infectious challenge is required to trigger antibody and T-cell based protective immunity to *H. polygyrus* (Hewitson et al., 2015), although it may require more challenges in wild mice. Because young adult and adult mice are likely to have previously experienced multiple helminth challenges, their baseline antibody levels were already established both pre- and post-treatment. In this case, mice are more likely to remain protected from re-infection following treatment. On the other hand, antibody levels in juvenile mice, and hence protective immunity, may not yet be established to a level where they are protective against re-infection following treatment. This result clearly demonstrates that age is crucial for the treatment success for an individual, and hence the distribution of ages in a population may impact the success or implementation of wider population-level treatment programmes and therefore should be taken into account in the design of treatment programmes.

Overall, our results provide a unique insight into the dynamic relationship between host immunity and parasite infection in the wild, which was only possible because we covered a wide span of demographic (age) variation. These findings suggest a need for alternative drug-independent interventions to prevent helminth infections in young individuals. For example, preventing extensive exposure in young individuals could serve as a tool to increase protection against high helminth burdens and increase the efficacy of disease treatment programmes. Our results highlight the unique insights we can gain into the factors shaping the immune environments of individuals in their natural habitats by incorporating traditional immunological approaches within ecological experiments.

## Funding

This work was supported by an Wellcome Trust ISSF grant to ABP and SAB [097821/Z/11/Z], a University of Edinburgh Torrance Bequest Scholarship to MC, grants from the National Environment Research Council to ABP and AF [NE/G006830/1, NE/G007349/1, NE/I024038/1 and NE/I026367/1], The Wellcome Trust [CIIE: 095831], a targeted Institute of Biodiversity, Animal Health & Comparative Medicine Research Fellowship to SAB, and a Chancellors Fellowship to ABP from the University of Edinburgh.

## Authors' contributions

MC, SAB and ABP coordinated and performed the field work. MC coordinated and performed the lab work. MC performed all analyses and wrote the manuscript. ABP, AF and SAB contributed to manuscript writing.

## Data accessibility

Data will be made available on Dryad.

## Conflicts of interest

Declarations of interest: none.

## References

[bib1] Albonico M., Allen H., Chitsulo L., Engels D., Gabrielli A.-F., Savioli L. (2008). Controlling soil-transmitted helminthiasis in pre-school age children through preventive chemotherapy. PLoS Neglected Trop. Dis..

[bib2] Babayan S.A., Allen J.E., Bradley J.E., Geuking M.B., Graham A.L., Grencis R.K., Kaufmann J., McCoy K.D., Paterson S., Smith K.G.C., Turnbaugh P.J., Viney M.E., Maizels R.M., Pedersen A.B. (2011). Wild immunology: converging on the real world. Ann. N. Y. Acad. Sci..

[bib3] Babayan S.A., Sinclair A., Duprez J.S., Selman C. (2018). Chronic helminth infection burden differentially affects haematopoietic cell development while ageing selectively impairs adaptive responses to infection. Sci. Rep..

[bib4] Behnke J., Harris P.D. (2010). Heligmosomoides bakeri: a new name for an old worm?. Trends Parasitol..

[bib5] Beura L.K., Hamilton S.E., Bi K., Schenkel J.M., Odumade O.A., Casey K.A., Thompson E.A., Fraser K.A., Rosato P.C., Filali-Mouhim A., Sekaly R.P., Jenkins M.K., Vezys V., Haining W.N., Jameson S.C., Masopust D. (2016). Normalizing the environment recapitulates adult human immune traits in laboratory mice. Nature.

[bib6] Bordon Y. (2016). Infection: go wild?. Nat. Rev. Immunol..

[bib7] Budischak S.A., Jolles A.E., Ezenwa V.O. (2012). Direct and indirect costs of co-infection in the wild: linking gastrointestinal parasite communities, host hematology, and immune function. Int. J. Parasitol. Parasites Wildl..

[bib8] Budischak S.A., Sakamoto K., Megow L.C., Cummings K.R., Urban J.F., Ezenwa V.O. (2015). Resource limitation alters the consequences of co-infection for both hosts and parasites. Int. J. Parasitol..

[bib9] Burlet P., Deplazes P., Hegglin D. (2010). Efficient age determination: how freezing affects eye lens weight of the small rodent species Arvicola terrestris. Eur. J. Wildl. Res..

[bib10] Cable J., Harris P.D., Lewis J.W., Behnke J.M. (2006). Molecular evidence that Heligmosomoides polygyrus from laboratory mice and wood mice are separate species. Parasitology.

[bib11] Cerutti A., Rescigno M. (2008). The biology of intestinal immunoglobulin A responses. Immunity.

[bib12] Clerc M., Devevey G., Fenton A., Pedersen A.B. (2018). Antibodies and coinfection drive variation in nematode burdens in wild mice. Int. J. Parasitol..

[bib13] Davis P.J., Parry S.H., Porter P. (1978). The role of secretory IgA in anti-coccidial immunity in the chicken. Immunology.

[bib14] DeVeale B., Brummel T., Seroude L. (2004). Immunity and aging: the enemy within?. Aging Cell.

[bib15] Dryden M.W., Payne P.A., Ridley R., Smith V. (2005). Comparison of common fecal flotation techniques for the recovery of parasite eggs and oocysts. Vet. Therapeut..

[bib16] Dunn P.K., Smyth G.K. (2008). Evaluation of Tweedie exponential dispersion model densities by Fourier inversion. Stat. Comput..

[bib17] Ebert D., Zschokke-Rohringer C.D., Carius H.J. (2000). Dose effects and density-dependent regulation of two microparasites of Daphnia magna. Oecologia.

[bib18] Esser-von Bieren J., Mosconi I., Guiet R., Piersgilli A., Volpe B., Chen F., Gause W.C., Seitz A., Verbeek J.S., Harris N.L. (2013). Antibodies trap tissue migrating helminth larvae and prevent tissue damage by driving IL-4Ralpha-independent alternative differentiation of macrophages. PLoS Pathog..

[bib19] Fenton A. (2013). Dances with worms: the ecological and evolutionary impacts of deworming on coinfecting pathogens. Parasitology.

[bib20] Ginaldi L., Loreto M.F., Corsi M.P., Modesti M., De Martinis M. (2001). Immunosenescence and infectious diseases. Microb. Infect..

[bib21] Griffiths E.C., Fairlie-Clarke K., Allen J.E., Metcalf C.J., Graham A.L. (2015). Bottom-up regulation of malaria population dynamics in mice co-infected with lung-migratory nematodes. Ecol. Lett..

[bib22] Hamm D.M., Agossou A., Gantin R.G., Kocherscheidt L., Banla M., Dietz K., Soboslay P.T. (2009). Coinfections with Schistosoma haematobium, Necator americanus, and Entamoeba histolytica/Entamoeba dispar in children: chemokine and cytokine responses and changes after antiparasite treatment. J. Infect. Dis..

[bib23] Hardy A.R., Quy R.J., Huson L.W. (1983). Estimation of age in the Norway rat (Rattus-Norvegicus Berkenhout) from the weight of the eyelens. J. Appl. Ecol..

[bib24] Hayward A.D., Garnier R., Watt K.A., Pilkington J.G., Grenfell B.T., Matthews J.B., Pemberton J.M., Nussey D.H., Graham A.L. (2014). Heritable, heterogeneous, and costly resistance of sheep against nematodes and potential feedbacks to epidemiological dynamics. Am. Nat..

[bib25] Hegewald J., Gantin R.G., Lechner C.J., Huang X., Agosssou A., Agbeko Y.F., Soboslay P.T., Kohler C. (2015). Cellular cytokine and chemokine responses to parasite antigens and fungus and mite allergens in children co-infected with helminthes and protozoa parasites. J. Inflamm..

[bib26] Hewitson J.P., Filbey K.J., Esser-von Bieren J., Camberis M., Schwartz C., Murray J., Reynolds L.A., Blair N., Robertson E., Harcus Y., Boon L., Huang S.C., Yang L., Tu Y., Miller M.J., Voehringer D., Le Gros G., Harris N., Maizels R.M. (2015). Concerted activity of IgG1 antibodies and IL-4/IL-25-dependent effector cells trap helminth larvae in the tissues following vaccination with defined secreted antigens, providing sterile immunity to challenge infection. PLoS Pathog..

[bib27] Humphreys N.E., Grencis R.K. (2002). Effects of ageing on the immunoregulation of parasitic infection. Infect. Immun..

[bib28] Jackson J.A., Hall A.J., Friberg I.M., Ralli C., Lowe A., Zawadzka M., Turner A.K., Stewart A., Birtles R.J., Paterson S., Bradley J.E., Begon M. (2014). An immunological marker of tolerance to infection in wild rodents. PLoS Biol..

[bib29] Knowles S.C., Fenton A., Pedersen A.B. (2012). Epidemiology and fitness effects of wood mouse herpesvirus in a natural host population. J. Gen. Virol..

[bib30] Knowles S.C., Fenton A., Petchey O.L., Jones T.R., Barber R., Pedersen A.B. (2013). Stability of within-host-parasite communities in a wild mammal system. Proc. Biol. Sci..

[bib31] Lagrue C., Poulin R. (2008). Intra- and interspecific competition among helminth parasites: effects on Coitocaecum parvum life history ory strategy, size and fecundity. Int. J. Parasitol..

[bib32] Laing R., Gillan V., Devaney E. (2017). Ivermectin - old drug, new tricks?. Trends Parasitol..

[bib33] Macpherson A.J., Geuking M.B., McCoy K.D. (2012). Homeland security: IgA immunity at the frontiers of the body. Trends Immunol..

[bib34] Maizels R.M., Hewitson J.P., Murray J., Harcus Y.M., Dayer B., Filbey K.J., Grainger J.R., McSorley H.J., Reynolds L.A., Smith K.A. (2012). Immune modulation and modulators in Heligmosomoides polygyrus infection. Exp. Parasitol..

[bib35] McCoy K.D., Stoel M., Stettler R., Merky P., Fink K., Senn B.M., Schaer C., Massacand J., Odermatt B., Oettgen H.C., Zinkernagel R.M., Bos N.A., Hengartner H., Macpherson A.J., Harris N.L. (2008). Polyclonal and specific antibodies mediate protective immunity against enteric helminth infection. Cell Host Microbe.

[bib36] Mestas J., Hughes C.C. (2004). Of mice and not men: differences between mouse and human immunology. J. Immunol..

[bib37] Montgomery W.I. (1989). Population regulation in the wood mouse, Apodemus sylvaticus. I. Density dependence in the annual cycle of abundance. J. Anim. Ecol..

[bib38] Morris P. (1972). A review of mammalian age determination methods. Mamm Rev..

[bib39] Nussey D.H., Watt K.A., Clark A., Pilkington J.G., Pemberton J.M., Graham A.L., McNeilly T.N. (2014). Multivariate immune defences and fitness in the wild: complex but ecologically important associations among plasma antibodies, health and survival. Proc. Biol. Sci..

[bib40] Nussey D.H., Watt K.A., Pilkington J.G., Zamoyska R., McNeilly T.N. (2012). Age-related variation in immunity in a wild mammal population. Aging Cell.

[bib41] Pedersen A.B., Babayan S.A. (2011). Wild immunology. Mol. Ecol..

[bib42] Reynolds L.A., Filbey K.J., Maizels R.M. (2012). Immunity to the model intestinal helminth parasite Heligmosomoides polygyrus. Semin. Immunopathol..

[bib43] Rowe F.P., Bradfield A., Quy R.J., Swinney T. (1985). Relationship between eye lens weight and age in the wild house mouse (Mus musculus). J. Appl. Ecol..

[bib44] Speich B., Moser W., Ali S.M., Ame S.M., Albonico M., Hattendorf J., Keiser J. (2016). Efficacy and reinfection with soil-transmitted helminths 18-weeks post-treatment with albendazole-ivermectin, albendazole-mebendazole, albendazole-oxantel pamoate and mebendazole.

[bib45] Tanikawa T. (1993). An eye-lens weight curve for determining age in black rats, *Rattus rattus*. J. Mammal. Soc. Jpn..

[bib46] Thomas R.E., Bellis E.D. (1980). An eye-lens weight curve for determining age in microtus-pennsylvanicus. J. Mammal..

[bib47] Trees A.J., Karim M.J., Mckellar S.B., Carter S.D. (1989). Eimeria tenella: local antibodies and interactions with the sporozoite surface. J. Protozool..

[bib48] Valanparambil R.M., Segura M., Tam M., Jardim A., Geary T.G., Stevenson M.M. (2014). Production and analysis of immunomodulatory excretory-secretory products from the mouse gastrointestinal nematode Heligmosomoides polygyrus bakeri. Nat. Protoc..

[bib49] Wahid F.N., Behnke J.M. (1993). Immunological relationships during primary infection with *Heligmosomoides polygyrus* (*Nematospiroides dubius*): parasite specific IgG1 antibody responses and primary response phenotype. Parasite Immunol..

[bib50] Wahid F.N., Behnke J.M., Conway D.J. (1989). Factors affecting the efficacy of ivermectin against *Heligmosomoides polygyrus* (*Nematospiroides dubius*) in mice. Vet. Parasitol..

[bib51] Wood S.N. (2011). Fast stable restricted maximum likelihood and marginal likelihood estimation of semiparametric generalized linear models. J. R. Stat. Ser. Soc. B Stat. Methodol..

